# Patient delay in oral cancer in North-Western Spain: Where do we stand?

**DOI:** 10.4317/medoral.28246

**Published:** 2026-06-28

**Authors:** Helena Sánchez Ortega, Pablo Varela Centelles, Juan Seoane, José Luis López-Cedrún, Arturo Bilbao Alonso, Lucía García Caballero, Andrés Blanco Hortas, Juan M. Seoane-Romero

**Affiliations:** 1 Oral & Maxillofacial Surgery Service, A Coruña University Hospital, Galician Health Service, A Coruña, Spain; 2 Praza do Ferrol Oral Healthcare Unit, Galician Health Service, Lugo, Spain; 3 Department of Surgery, School of Medicine and Dentistry, University of Santiago de Compostela, A Coruña, Spain; 4 DePaCra Research Group, Santiago de Compostela Investigation Institute, Santiago de Compostela, A Coruña, Spain; 5 Oral & Maxillofacial Surgery, Santiago de Compostela University Hospital, Santiago de Compostela, A Coruña, Spain; 6 Department of Morphological Sciences, School of Medicine and Dentistry, University of Santiago de Compostela, Santiago de Compostela, Spain; 7 Santiago de Compostela Investigation Institute, Lugo, Spain

**Keywords:** Oral cancer, diagnostic intervals, patient interval, early diagnosis, time-to-treatment, Aarhus Statement.

## Abstract

**Background:**

Timely diagnosis remains a major challenge in oral cancer, with more than half of cases diagnosed at advanced stages. Although the patient interval has been suggested as a key component of the diagnostic pathway, its relative contribution and temporal evolution remain insufficiently characterized. This study aimed to quantify diagnostic and treatment intervals in oral cancer and to evaluate the relative contribution of the patient interval using the Aarhus Statement framework, comparing current data with a historical cohort recruited a decade earlier in North-Western Spain.

**Material and Methods:**

A hospital-based ambispective observational study was conducted including consecutive incident cases of histologically confirmed oral squamous cell carcinoma diagnosed in 2025 at a tertiary referral center. Time intervals from first symptom to treatment initiation were defined according to the Aarhus Statement. Recall bias was minimized through triangulation of patient interviews, relatives’ reports, and clinical records. Median intervals and interquartile ranges were calculated, and comparisons were performed with a historical cohort recruited in 2015 under identical methodological criteria. Ratios between intervals were estimated to assess the relative contribution of the patient interval.

**Results:**

The median patient interval decreased slightly in 2025 compared with 2015 (25 vs. 31.5 days). Primary care and diagnostic intervals were shorter, whereas specialist and pre-treatment intervals increased. The total pre-treatment interval remained stable between cohorts. Ratio analyses showed that the patient interval represented a limited proportion of the total diagnostic and treatment pathway, with no significant temporal differences.

**Conclusions:**

Temporal changes in the oral cancer pathway were heterogeneous, suggesting a redistribution of delays toward later intervals of care. The patient interval was not the main contributor to overall time to treatment, highlighting the need for system-level strategies targeting specialist assessment and treatment initiation phases.

## Introduction

Oral cancer was the 16th most common malignancy worldwide in 2022, with marked international variability in incidence rates [1]. The highest incidence has been reported in Palau and several Asian regions. Countries such as India, Austria, the Czech Republic, and the United Kingdom have also experienced rising incidence trends in recent years [2,3]. In this context, lip and oral cavity cancers represent a major public health challenge in Europe, with 62,103 incident cases reported in 2022 [4]. In Spain specifically, a 35.4% increase in incident cases and a 42.3% increase in mortality are estimated by 2045 [1].

More than half of oral cancer cases are diagnosed at advanced stages (stage III-IV), which substantially compromises prognosis. Five-year survival rates range between 30% and 65% and have shown little improvement over recent decades [4-6]. 

Early diagnosis and timely treatment are associated with improved quality of life and better overall survival, although the strength of this association varies across cancer types [7]. In oral cancer, patients experiencing diagnostic delays are significantly more likely to present with advanced-stage disease [8], and such delays constitute a moderate risk factor for cancer-related mortality in head and neck cancers [9].

Although the term diagnostic delay has been recognized as potentially stigmatizing-due to its implicit attribution of “culpability” and its medico-legal implications-it has been widely adopted in the scientific literature for over 80 years [10]. This historical usage has led to substantial heterogeneity in conceptual definitions and criteria for diagnostic delay, hindering evidence synthesis and comparability across studies [11,12]. Consequently, its use has been discouraged in favor of describing specific events, processes, time intervals, and contributing factors within a structured model of the cancer patient pathway to treatment [11]. Adoption of this conceptual framework has improved the design and reporting quality of studies on early cancer diagnosis [11,13].

Despite evidence indicating that the patient interval constitutes the largest component of the diagnostic pathway in oral cancer [14], and their potential association with advanced-stage at diagnosis [15,16], this interval remains scarcely studied.

The aim of this study was to quantify the duration of the time intervals elapsed until initiation of treatment, focusing on the relative contribution of the patient interval compared with the other intervals defined in the Aarhus Statement. Additionally, we sought to evaluate temporal trends over the past decade by comparing current findings with a historical 2015-cohort recruited using identical methodological criteria [17-19]. 

## Materials and Methods

A hospital-based observational study with an ambispective design was conducted. The prospective component commenced at the time of the patient’s first contact with the specialist responsible for treatment. A convenience sample was considered, consisting of the consecutive recruitment of incident cases of patients with histologically confirmed oral squamous cell carcinoma diagnosed at the Oral and Maxillofacial Surgery Service of CHUAC (A Coruña, Spain) (2025 cohort: Participation rate 98%). Prevalent or recurrent cases, second primary tumors, multiple carcinomas, and cases diagnosed outside the study setting were excluded.

Within the framework of the Aarhus Statement, all overall milestones and time intervals along the pathway from first symptom to initiation of treatment were assessed. Specifically, the following intervals were defined: 

Patient interval: From first symptom to first presentation or clinical appearance.

Primary care interval: From first presentation to first referral to secondary care.

Specialist interval: From first contact with the specialist to diagnosis.

Diagnostic interval: From first presentation to histological diagnosis. 

Pre-treatment (treatment) interval: From diagnosis to start of treatment. 

Also, the following composite intervals were analyzed: 

Pre-referral interval: Patient interval plus primary care interval. 

Total diagnostic interval: From first symptom (detection of bodily change) to date of diagnosis. 

Total pre-treatment (treatment) interval: From first symptom to start of treatment.

To minimize recall bias, the study incorporated triangulation strategies whereby patient-reported information was cross-checked against reports from family members and verified using clinical records. To evaluate the relative contribution of the patient interval within the oral cancer care pathway, the ratio of the patient interval to each subsequent interval up to treatment initiation was calculated. Finally, the duration of all in-tervals in the current cohort was compared with those of a historical cohort recruited 10 years earlier, within the same clinical setting and under identical inclusion and exclusion criteria (2015 cohort) [17]. All patients provided written informed consent prior to study entry. The study protocol was approved by the institutional Research Ethics Committee (approval No. 2021/427).

*Statistical analysis*

For the statistical analysis, the mean and median were used as measures of central tendency, and the interquartile range was employed as a measure of dispersion. 

Comparison of median ratios (from reported medians and 95% confidence intervals): For time intervals, where only medians and interquartile ranges (IQR) were available, comparisons between cohorts were performed on the logarithmic scale to account for skewness and the strictly positive distribution of time variables. The standard error of each median was approximated using the IQR and the corresponding sample size (actual number of non-missing observations per interval) based on large-sample assumptions. Log-scale differences and their 95% confidence intervals were estimated assuming independence between cohorts and were subsequently back-transformed to express results as ratios of medians. These estimates should be interpreted as approximate, as they rely on summary statistics rather than individual-level data.   All statistical analyses were performed using R version 4.3.3 (R core Team 2023).

**Results** 

In the cohort recruited in 2025, a total of 118 patients were included: 61 males (51.7%) and 57 females (48.3%), with a mean age of 67.6±13.6 years. Disease TNM-stage distribution was as follows: 45 patients in stage I (38.1%), 20 in stage II (16.9%), 18 in stage III (15.3%), and 35 in stage IV (29.7%). Oral squamous cell carcinomas (OSCC) were predominantly located in the tongue (n=46; 39%), followed by the gingiva (n=26; 22.0%), floor of mouth (n=15; 12.8%), palate (n=7; 5.9%), and other anatomical sites (n=24; 20.3%).

Meanwhile, the historical cohort comprised 74 patients, predominantly male (59.5%), with a median age of 65 years (IQR: 57-74). Most cases were diagnosed at advanced stages (TNM III-IV: n=39; 52.7%). The tongue was again the most frequent tumor site (n=38; 51.4%), followed by the palate (n=10; 13.5%), floor of mouth/gingiva (n=4; 5.4%), and other locations (n=17; 23%). The median patient interval was slightly shorter in 2025 compared with 2015 historical cohort (25; IQR:8.5-73.5 vs. 31.5; IQR: 7.00-61.00 days), although the mean values were comparable (50.6 vs. 45.9 days). The primary care interval demonstrated a marked reduction in the 2025 cohort, with a median of 1 day (IQR: 0-10) compared with 6.5 days (IQR: 0.00-49-25) in 2015. Similarly, the pre-referral interval decreased in 2025 (median 44; IQR:19.75-95 vs. 66.5 days; IQR:39.00-129.00).

In contrast, the specialist interval was longer in the 2025 cohort, with a median of 10 days (IQR: 6.00-15.00) compared with 6 days (IQR: 4.00-11.25) in the historical cohort. The diagnostic interval also showed a reduction over time, with a median of 24 days (11.00-52.00) in 2025 versus 36 days (IQR: 12.00-86.00) in 2015, resulting in a shorter total diagnostic interval (median 77; IQR: 35.00-125 vs. 86; IQR:35.50-146.25 days).

Conversely, the pre-treatment interval increased substantially in the 2025 cohort (median 41; IQR: 28-63 vs. 22 days (IQR:14.25-33.00), while the total pre-treatment interval remained broadly comparable between cohorts (median 117; IQR: 80.00-177.00 vs. 103 days; IQR:61.75-176.00), despite higher upper percentiles observed in the 2025 cohort.  Compared with the 2015 cohort, the 2025 cohort showed shorter median intervals for primary care, pre-referral, diagnostic, and total diagnostic phases. However, longer delays were observed in the specialist and pre-treatment intervals, with the increase in the specialist interval reaching statistical significance. Overall, the total pre-treatment interval remained similar between cohorts, although greater variability was observed in the 2025 cohort (Table 1, Table 2).

When expressed as ratios with respect to broader phases of care, the patient interval showed median ratios of 0.70 (IQR: 0.26-0.96) in the 2015 cohort and 1.00 (IQR: 0.81-1.00) in the 2025 cohort relative to the pre-referral interval. Lower ratios were observed when the patient interval was compared with the total diagnostic interval (median 0.37 in 2015 and 0.43 in 2025) and with the total pre-treatment interval (median 0.26; IQR: 0.07-0.41 and 0.21; IQR:0.13-0.30, respectively) (Figure 1; Table 3). 

When ratios were calculated between the patient interval and other discrete intervals, different patterns were observed. Relative to the primary care interval, the patient interval was longer in the 2015 cohort (median ratio 1.25; IQR: 0.05-7.71), whereas the inverse relationship was observed in the 2025 cohort (median ratio 0.66; IQR:0.23-1.85). In comparison with the diagnostic interval, mean ratios were higher in the 2025 cohort, although median ratios remained below 1 in both cohorts. Similarly, ratios between the patient and pre-treatment intervals were consistently below 0.3 across cohorts (Table 3).

## Discussion

In this study, we analysed temporal changes in the patient pathway to treatment by comparing two cohorts separated by a decade, using complementary approaches based on absolute time intervals, relative ratios between intervals, and formal comparisons of medians between cohorts. This multifaceted strategy allowed us to characterise not only whether time to treatment changed over time, but also how different components of the pathway contributed to the overall timeline. 

This is the first study in oral cancer to adopt a conceptual framework-the Aarhus Statement-and to longitudinally examine the evolution of each diagnostic time interval and their relative contribution to the overall time interval. To enhance internal validity, specific strategies were implemented to minimize recall bias, and the high participation rate makes selection bias unlikely. Nevertheless, as this is a hospital-based, single-center study, limitations regarding the potential extrapolation of the findings should be acknowledged. However, Spain benefits from a universal, publicly funded healthcare system with a relatively homogeneous secondary care structure, which may partially mitigate concerns related to generalizability.

The median patient interval observed in the 2025 cohort was shorter than that reported in a recent quantitative synthesis, which estimated a pooled patient interval of 47 days (95% CI: 31-73) and was also lower than the estimate reported for high-income countries (30 days, 95% CI: 23-53) [18]. This finding suggests a comparatively earlier patient response in the present cohort. Nevertheless, the patient interval remains one of the most under-researched components of the diagnostic pathway, particularly with regard to the appraisal and help-seeking phases [11,19]. Previous studies have attributed delays during this interval to the absence of pathognomonic signs and symptoms, misinterpretation or misattribution of early manifestations, limited health literacy, and broader socio-cultural factors influencing symptom perception and care-seeking behaviour [19,20]. Despite its intuitive relevance, evidence regarding the prognostic impact of the patient interval remains inconsistent. Prior research has reported conflicting associations between patient delay and tumour stage at diagnosis [16,21,22], and its effect on patient survival remains largely unknown [18]. Taken together, these findings underscore the complexity of the patient interval and highlight the need for further research to clarify its determinants and clinical significance within the broader diagnostic and therapeutic pathway.

The duration of the primary care interval has decreased substantially over the past decade, currently reaching a mean of 19.6 days. This figure is comparable to that reported in the United Kingdom [23], and lower than those described in the United States by Peacock *et al*. [24]. Nevertheless, considerable variability persists across countries, largely reflecting differences in the organization and structure of their healthcare systems [25]. In addition, referral patterns-specifically, referrals initiated by General Dental Practitioners (GDPs) versus General Practitioners (GPs)-appear to influence the length of this interval. Although some studies have failed to identify significant differences in diagnostic delay according to the type of primary healthcare provider, others have shown that GDPs are associated with longer intervals to diagnosis and require a higher number of consultations before referring patients to secondary care [22]. The number of consultations prior to referral therefore constitutes a surrogate marker of the primary care interval [26]. On average, patients require two to three consultations before being referred from dental care to secondary healthcare services [27]. This delay is likely related to the use of “treatment trials,” such as the removal of local irritative factors-including poorly fitting dentures-before specialist referral is considered [27].

The specialist time interval (STI) is relatively short in the diagnostic pathway of oral cancer and represents a limited proportion of the overall time elapsed until treatment initiation. However, we have identified a concerning increase in the duration of this period. This finding suggests that there is still room for improvement and that the STI may constitute a potential target for future interventions aimed at shortening diagnostic delays, particularly among patients diagnosed at early stages following disease disclosure [28].

Our study also documents a substantial reduction in the diagnostic interval over the past decade. Although the prognostic impact of diagnostic delay remains to be fully elucidated, the existing evidence continues to be controversial [8,9,29]. These inconsistencies are likely attributable to the lack of an appropriate conceptual framework and to methodological weaknesses that have been frequently identified in this type of research [8,11].

The pre-treatment interval, defined as the time between histological diagnosis and initiation of treatment, has shown a concerning increase over the past decade. This trend is particularly worrisome given that prolonged pre-treatment intervals have been consistently associated with adverse survival outcomes, including poorer overall and disease-specific survival, as well as reduced postoperative quality of life [30].

Our results show that changes in the patient pathway were heterogeneous rather than uniform. Several early intervals, including the patient, primary care, and pre-referral intervals, showed shorter median durations in the 2025 cohort compared with the 2015 cohort. These descriptive findings suggest a potential temporal shift toward earlier engagement with the healthcare system and more streamlined referral processes. However, formal comparisons of medians indicated that most of these reductions did not reach statistical significance, highlighting the considerable variability inherent in time-to-event data and underscoring the importance of cautious interpretation. 

In contrast, later intervals of the pathway displayed a different pattern. Both the specialist interval and the pre-treatment interval were significantly longer in the 2025 cohort. This finding suggests that, despite potential improvements in early phases of care, delays may have shifted toward later stages, particularly after specialist assessment and before treatment initiation. Importantly, these opposing trends help explain why the total pre-treatment interval remained broadly stable between cohorts, despite notable changes in individual components. This apparent stability reflects a redistribution of delays along the pathway rather than a global reduction in time to treatment.

## Conclusions

The analysis of ratios between the patient interval and other intervals provided additional insight into the internal structure of the patient pathway and helped to identify priority areas for health policy and system-level interventions. Across both cohorts, the patient interval consistently accounted for a smaller share of the total diagnostic and total pre-treatment intervals, indicating that patient-related delay was not the dominant contributor to overall time to treatment. Moreover, comparisons of these ratios between cohorts revealed no statistically significant differences, suggesting that the relative role of the patient interval within the broader pathway remained largely unchanged over time, even as absolute durations of certain intervals varied. 

## Figures and Tables

**Table 1:** Table 1: Time intervals (days) until treatment start.

	**2025 cohort**					**2015 cohort**				
**Intervals (days)**	**Mean**	**25P**	**50P**	**75P**	**90P**	**Mean**	**25P**	**50P**	**75P**	**90P**
**Patient interval**	50.60	8.50	25.00	73.5	128.60	45.86	7.00	31.50	61.00	121.40
**Primary care interval**	19.64	0.00	1.00	10.00	71.20	29.08	0.00	6.50	49.25	74.00
**Pre-referral interval**	67.35	19.75	44.00	95.00	142.50	83.61	39.00	66.50	129.00	168.90
**Specialist interval**	22.40	6.00	10.00	15.00	36.00	11.54	4.00	6.00	11.25	22.60
**Diagnostic interval**	44.86	11.00	24.00	52.00	116.40	63.20	12.00	36.00	86.00	133.00
**Total diagnostic interval**	92.63	35.00	77.00	125.00	184.20	103.23	35.50	86.00	146.25	195.60
**Pre-treatment interval**	59.46	28.00	41.00	63.00	108.80	28.75	14.25	22.00	33.00	40.00
**Total pre-treatment interval**	142.2	80.00	117.00	177.00	264.60	130.80	61.75	103.00	176.00	246.50

P: Percentile.

**Table 2:** Table 2: Comparison of median intervals (days) and the difference in median ratios across the studied cohorts.

**Interval**	**Cohort 2015**	**Cohort 2025**	**Ratio of medians**	**p-**
	Median (IQR)	Median (IQR)	(95%CI)	
Patient interval	31.5 (7-61)	25 (8.5-73.5)	0.794 (0.421-1.496)	0.475
Primary care interval	6.5 (0-49.25)	8 (3-16)	1.231 (0.212-7.137)	0.817
Pre-referral interval	66.5 (39-129)	44 (19.75-95)	0.662 (0.415-1.054)	0.082
Specialist interval	6 (4-11.25)	10 (6-15)	1.667 (1.208-2.299)	0.002
Diagnostic interval	36 (12-86)	24 (11-52)	0.667 (0.376-1.182)	0.165
Total diagnostic interval	86 (35.5-146.25)	77 (35-125)	0.895 (0.623-1.287)	0.551
Pre-treatment interval	22 (14.25-33)	41 (28-63)	1.864 (1.447-2.399)	0
Total pre-treatment interval	103 (61.75-176)	117 (80-177)	1.136 (0.843-1.531)	0.402

**Table 3:** Table 3: Mean and median ratios of the patient interval to other time intervals (2015 and 2025 cohorts).

	**2015 cohort**		**2025 cohort**	
	**Mean**	**Median**	**Mean**	**Median**
	**(95% CI)**	**(95% CI)**	**(95% CI)**	**(95% CI)**
**Patient interval/Primary care interval**	5.12 (0.99-11.25)	1.25 (0.05-7.71)	11.38 (2.22-20.54)	0.659 (0.23-1.85)
**Patient interval/Pre-referral interval**	0.61 (0.43-0.78)	0.7 (0.26-0.96)	0.71 (0.626-0.80)	1 (0.81-1)
**Patient interval/Diagnostic interval**	1.91 (0.89-3.02)	0.79 (0.33-3.1)	3.29 (2.08-4.50)	0.73 (0.41-1.66)
**Patient interval/Total diagnostic interval**	0.4 (0.27-0.53)	0.37 (0.14-0.65)	0.45 (0.37-0.52)	0.43 (0.27-0.61)
**Patient interval/Pre-treatment interval**	0.3 (0.18-0.42)	0.26 (0.07-0.41)	0.26 (0.2-0.32)	0.21 (0.13-0.30)

**Figure 1:**
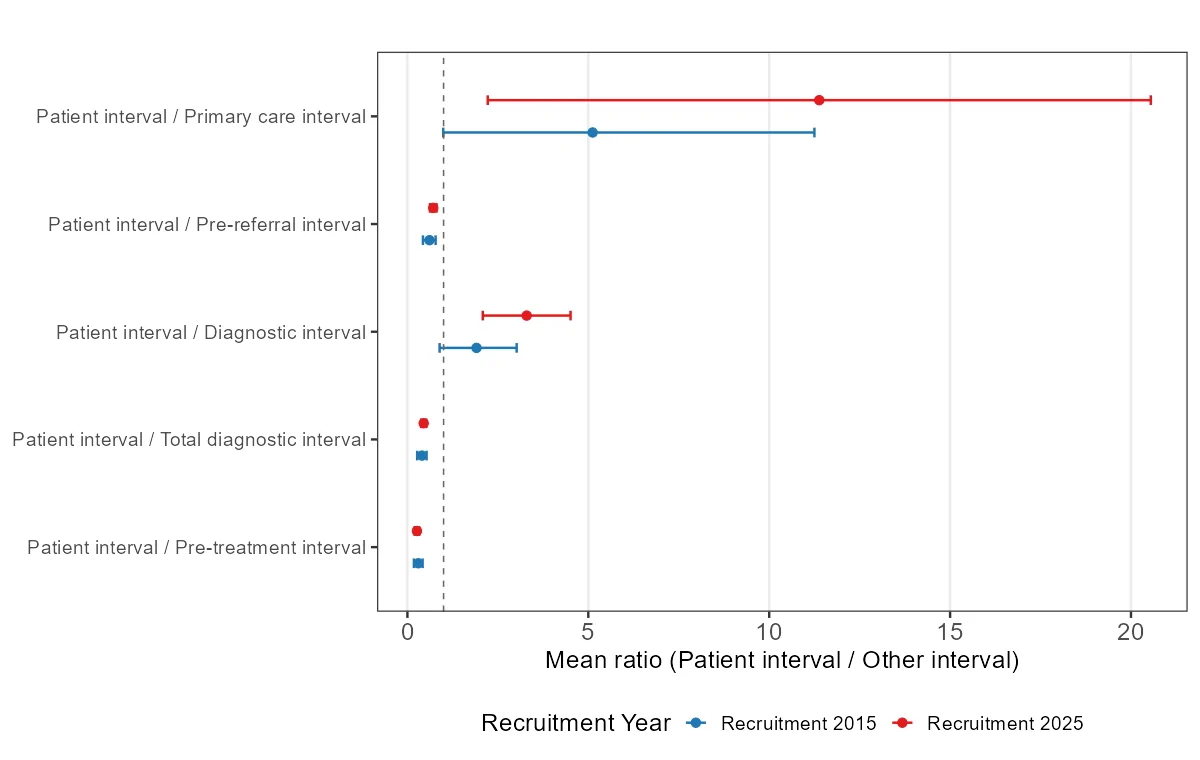
Fig. 1: Mean ratio of the patient interval relative to other time intervals (2015-2025 cohort).

## Data Availability

None.
